# Humidifier disinfectant, sodium dichloroisocyanurate (NaDCC): assessment of respiratory effects to protect workers’ health

**DOI:** 10.1038/s41598-021-95148-7

**Published:** 2021-08-03

**Authors:** DongSeok Seo, Yong-Hoon Lee, JiMin Jo

**Affiliations:** Inhalation Toxicity Study Center, Occupational Safety and Health Research Institute, Korea Occupational Safety and Health Agency, 30, Expro-ro 339beon-gil, Yuseong-gu, Daejeon, Republic of Korea

**Keywords:** Health occupations, Screening

## Abstract

In South Korea, it has been found that biocides used to control and eliminate harmful organisms are used as humidifier disinfectants and cause lung disease in users. Hence, efforts have been focused on studying the toxicity of biocides in workers who handle them. The purpose of this study was to evaluate the effects of inhalation exposure to sodium dichloroisocyanurate (NaDCC) to protect the health of workers handling NaDCC. F344 rats were exposed to 0.8-, 4-, and 20-mg/m^3^ of NaDCC for 6 h per day, 5 days per week for 14 days, and the recovery period after exposure was 14 days. In the 20-mg/m^3^-exposure group, we observed a decrease in food intake in females, a weight loss in males, and a decrease in partially active thromboplastin time in males and females 2 weeks after exposure. We noted a decrease in white blood cells in males in the 4- and 20-mg/m^3^-exposed groups. Both males and females in the 20-mg/m^3^ group and males in the 4-mg/m^3^ group showed irritation in the larynx related to test substance exposure. However, these findings were not observed in the recovery group. The main target organs affected by repeated 2-week inhalation exposure to NaDCC were the nasal cavity and larynx in the upper respiratory tract. The No Observed Adverse Effect Level (NOAEL) was considered to be 0.8 mg/m^3^ because effects related to NaDCC exposure were observed even at of 4 mg/m^3^, and these effects were found to be reversible.

## Introduction

According to the Biocides Directive (98/8/EC)^1^, biocides are active substances or preparations that are intended to destroy, deter, render harmless and exercise control or prevent the action of any other harmful organism through chemical or biological means. Biocides give example of the 23 product types, organized into several subgroups. Biocides are used because of their potential to destroy a wide range of organisms and for their relatively easy applicability to vessels^2^ and aquaculture systems. A biocidal product contains one or more of these biocidal substances. Chlorine has been used as a disinfectant for the treatment of drinking water for over 100 years. It is the most commonly used means of disinfecting water^3^, and its effectiveness as a microbicide has been widely assessed^4^. Sodium dichloro isocyanurate (NaDCC) is the sodium salt of 1,3-dichloro-1,3,5-triazine-2,4,6(lH,3H,5H)-trione and is a synthetic organic chlorine donor derived from isocyanurate. It is a white crystalline or granular powder of molecular weight 219.9 containing approximately 62% of ‘available chlorine’^5^. Most conventional systems in developed countries treat water as chlorine dioxide (delivered as a liquid in pressurized systems), but other common alternatives include calcium hypochlorite, sodium hypochlorite, lithium hypochlorite and chloro-isocyanurates (sodium dichloroisocyanurate or trichloroisocyanuric acid). Until recently, the isocyanurate was used chiefly in the disinfection of water for swimming pools and industrial cooling towers, and is also a common microbial agent in cleaning and disinfection applications including baby bottles and contact lens^6^.

All of these biocides disinfect water by releasing free available chlorine (FAC) in the form of hypochlorous acid (HOCl). For example,

$$ \begin{aligned} & \mathop {{\text{NaOCl}} + {\text{H}}_{{2}} {\text{O}}}\limits_{{\text{Sodium hypochlorite}}} \, \to \mathop {{\text{HOCl}} + {\text{NaOH}}}\limits_{{\text{dispersion in water}}} \\ & \mathop {{\text{NaCl}}_{{2}} ({\text{NCO}})_{{3}} + {\text{2H}}_{{2}} {\text{O}} \leftrightarrow {\text{2HOCl}} + {\text{NaH}}_{{2}} ({\text{NCO}})_{{3}} }\limits_{{\text{NaDCC dissolution in water}}} \\ \end{aligned} $$

FAC (chlorine in the + 1 oxidation state) is an effective biocide against a wide range of bacteria, fungi, algae, and viruses^7^. Solutions of hypochlorous acid (HOCl)/hypochlorite (ClO^-^) have excellent oxidizing and disinfecting properties. Hypochlorite is a strong oxidizing agent and is highly effective in eliminating organic contaminants, whereas undissociated HOCl is the principal microbiocidal agent that is effective against bacteria, fungi, algae, viruses, and other microorganisms^8,9^.

These biocides are used for a variety of purposes, and especially in South Korea, these biocides were used as effective ingredients for humidifier disinfectants in 31 products from 1994 to clean and disinfect the inside of humidifiers. The number of victims by the use of humidifier disinfectant was 5790 (as of October 23, 2017), of which 1256 (21.7%) died. This cause is thought to be the effect of the inhalation exposure of the humidifier disinfectant. NaDCC is also currently used in various industries, and the circulation amount of NaDCC in South Korea in 2016 was estimated to be approximately 200 kg according to the Ministry of Environment's press release. From 2005 to 2011, 36,850 units of humidifier disinfectants using NaDCC as an active ingredient were sold, and 89 victims were identified according to the Seoul Central District Prosecutor's Office press release (July 23, 2019). The fact that these biocides have been a problem for consumers in the living environment is thought to cause more serious problems for workers who manufacture or handle them. For example, According to NIOSH's (National Institute for Occupational Safety and Health) 1982 Health Risk Assessment Report, the concentration range for each particle in the finishing and packaging processes of TCCA (trichloroisocyanuric) and NaDCC ranged from 0.11 to 38 mg/m^3^ and the average was 2.4 mg/m^3^. Near the packing area, approximately 60% of the dust sampled by NIOSH was within the respirable size range (< 10 um aerodynamic equivalent diameter)^10^. To date, exposure criteria and recommended limits for NaDCC have not been developed, and toxicity data from inhalation exposure are very limited. Recently, due to the COVID 19 pandemic, it has been widely used as a variety of disinfectants. In addition, biocides are used for disinfection and cleaning of medical facilities with a high risk of infection, so the exposure of related workers is expected to be high.

All chlorine products have some level of toxicity that confers microbicidal efficacy. However, when chlorinated water is ingested, the available chlorine is rapidly reduced by saliva and stomach fluid to harmless chloride ions salts^11^. The unique characteristic of isocyanurates is cyanuric acid, the carrier that allows the chlorine to be contained in a solid, stable, and dry form. Because of the potential toxicity of cyanuric acid, NaDCC for routine treatment of drinking water requires regulatory review prior to approval. United States Environmental Protection Agency (US EPA) has approved the use of NaDCC as a hard-surface disinfectant for hospitals and manufacturing facilities when used in accordance with the label^12,13^. The toxicity of NaDCC and cyanuric acid have been extensively studied and documented in support of the registration of isocyanurates with the US EPA. These have been summarized in other reports^14,15^. Studies performed on acute toxicity and irritancy were intended to assess the safety of handling the dry product. These studies found chlorinated isocyanurates no more than slightly toxic and not corrosive. Chronic and sub-chronic toxicity studies also showed no toxicity. Developmental toxicity studies have also established that the compound is not fetotoxic, teratogenic (causing birth defects), mutagenic, or carcinogenic. Chlorinated isocyanurates are not accumulated or metabolized in the body^16^. However, there are no toxicological data for the inhalation exposure route. Hence, it is necessary to assess the hazards-risks, including biocide exposure assessments in South Korea to protect the health of workers in biocide-treated workplaces. Therefore, this study was conducted to evaluate the effects of respiratory exposure by inhalation on NaDCC, which may cause health risk for workers in their work environment.

## Results

**Figure 1:**
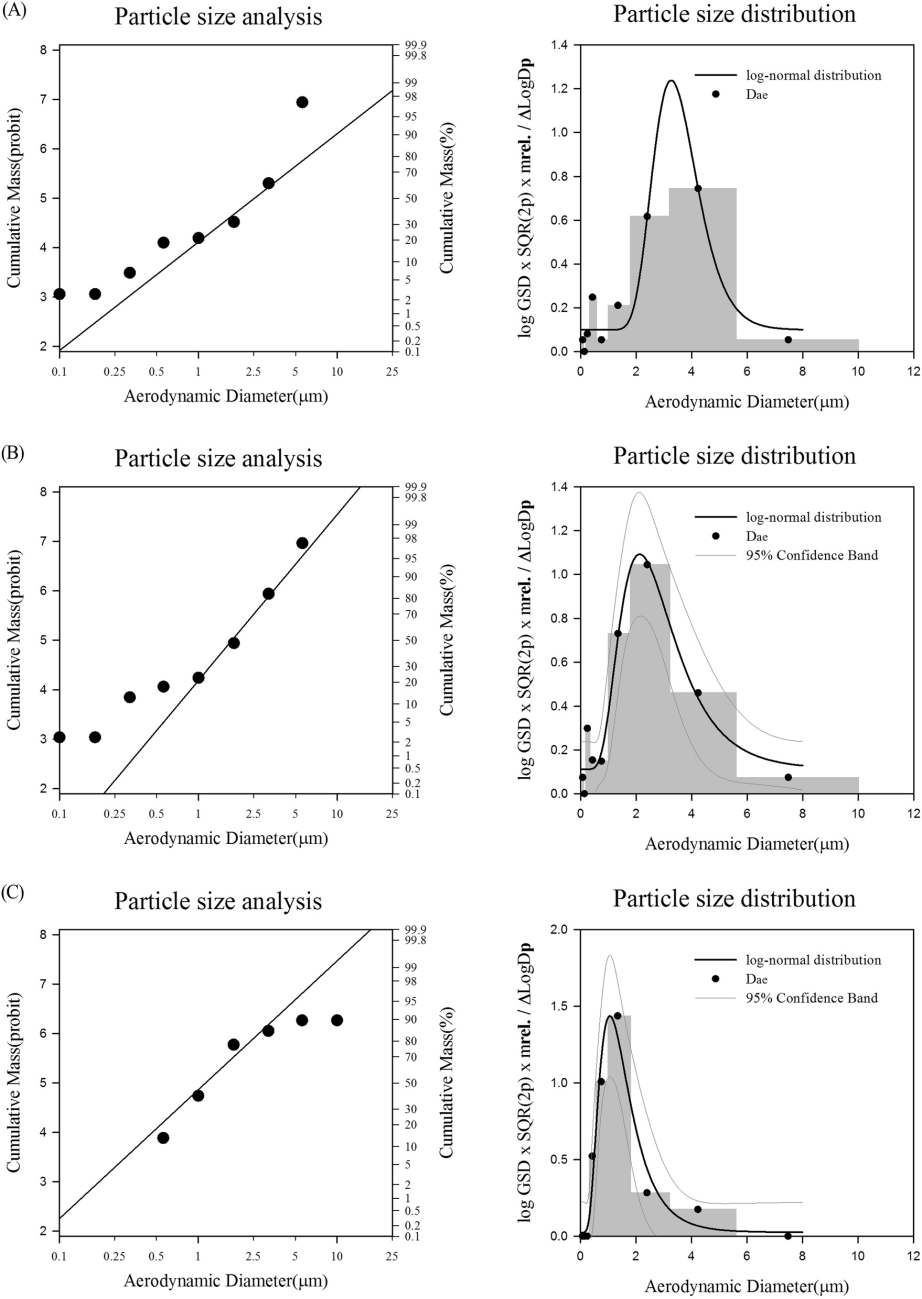
Particle size distributions of NaDCC in the chambers. 0.8 mg/m^3^ (**A**), 4.0 mg/m^3^ (**B**), 20 mg/m^3^ (**C**).

The average concentrations of NaDCC during exposure to 0.8, 4, and 20 mg/m^3^ were determined to be 0.82 ± 0.04, 3.83 ± 0.23, and 19.35 ± 1.01 mg/m^3^, respectively. During aerosol generation of 0.8, 4, and 20 mg/m^3^ of the test substance, the MMADs of the aerosols were determined to be 2.42, 1.87, and 1.13 μm, and the GSDs were 1.60, 1.75, and 1.41, respectively (Fig. 1). T_95_, the time to reach 95% of the target concentration in the chamber, was found to be 17.5, 10.9, and 2.2 min, respectively.

**Figure 2:**
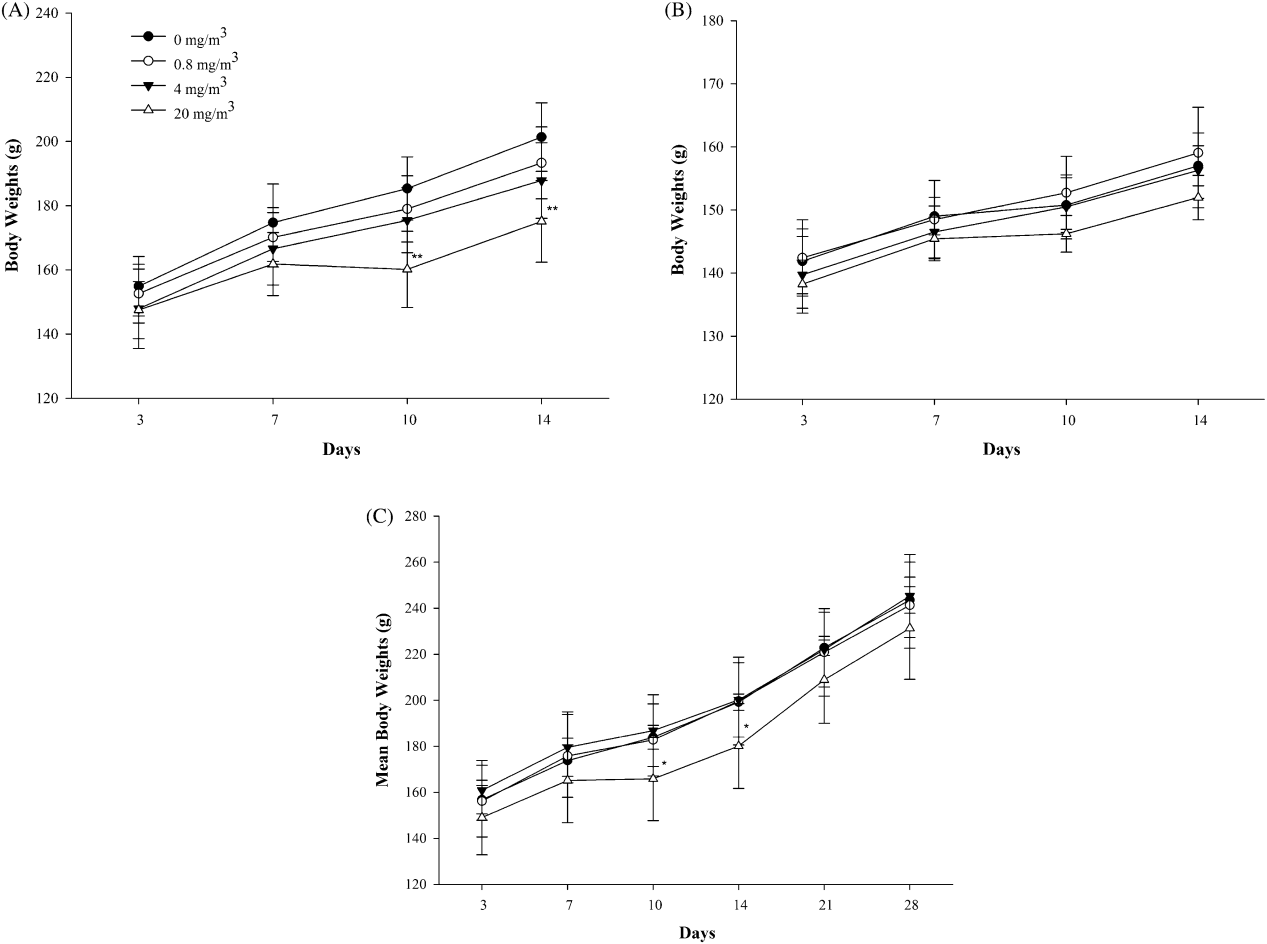
Changes of body weights in the rats exposed to NaDCC. Males (**A**) and Females (**B**) of the main groups, Males (**C**) of the recovery groups. Significantly different from control by Dunnett LSD test: *p < 0.05; **p < 0.01.

**Figure 3:**
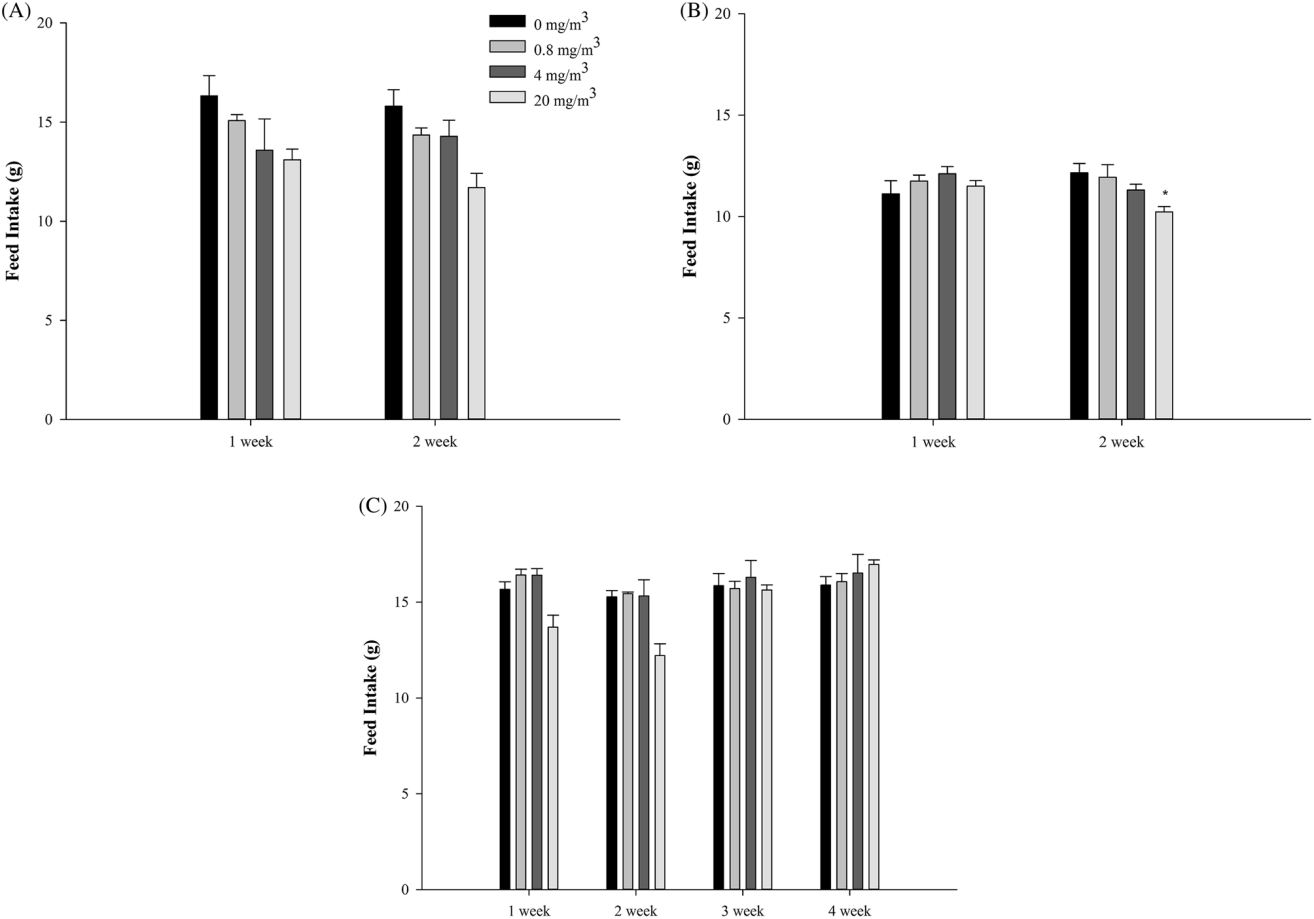
Changes of feed intake in the rats exposed to NaDCC. Males (**A**) and Females (**B**) in the main groups, Males (**C**) in the recovery groups. Significantly different from control by Dunnett LSD test: *p < 0.05.

No death or adverse clinical signs were observed in any test group during the test period. During the exposure period, significant weight loss was observed in the males of the 20-mg/m^3^ exposure group compared to the control group at 10 and 14 days after exposure. There was no significant weight change in the 0.8- and 4-mg/m^3^-exposure and recovery groups (Fig. 2). Food intake in females exposed to 20 mg/m^3^ at 2 weeks after initiation of exposure was significantly reduced compared to control group (Fig. 3), with no other significant changes.

In males, a significant change was observed in WBC counts and PT in the 20-mg/m^3^-exposure group compared to the control group, whereas only WBC counts were significantly lower in the 4-mg/m^3^-exposure group than in the control group. In females, a significant increase was observed in the PT of the 20-mg/m^3^-exposure group compared to the control group, and significant higher basophilic (BASO) counts were observed in the 4-mg/m^3^-exposure group than in the control group. In the recovery group, a significant increase was observed only in the PT of the 20-mg/m^3^-exposure group (Table 1).

**Table 1:** Hematological parameters of main group rats exposed to NaDCC.

Sex	Male				Female			
Concentration (mg/m^3^)	0	0.8	4	20	0	0.8	4	20
WBC (× 10^3^/µL)	4.33 ± 0.43	3.51 ± 0.83	3.22 ± 0.65	3.07 ± 0.65	2.99 ± 0.84	2.65 ± 0.36	2.61 ± 0.42	2.28 ± 0.42
RBC (× 10^6^/µL)	8.21 ± 0.21	8.21 ± 0.16	8.21 ± 0.17	7.99 ± 0.66	8.62 ± 0.26	8.65 ± 0.26	8.53 ± 0.25	8.41 ± 0.10
Hb (g/dL)	14.90 ± 0.12	15.02 ± 0.33	14.92 ± 0.19	14.64 ± 1.03	15.46 ± 0.58	15.42 ± 0.34	15.20 ± 0.35	15.06 ± 0.15
HCT (%)	43.08 ± 0.99	42.88 ± 0.69	42.48 ± 0.40	41.28 ± 3.32	43.22 ± 1.27	43.46 ± 1.15	42.82 ± 1.20	42.24 ± 0.45
MCV (fL)	52.50 ± 0.62	52.26 ± 0.41	51.72 ± 0.75	51.68 ± 0.16	50.14 ± 0.40	50.22 ± 0.44	50.20 ± 0.60	50.20 ± 0.21
MCH (g/dL)	18.16 ± 0.49	18.26 ± 0.30	18.16 ± 0.31	18.36 ± 0.33	17.92 ± 0.29	17.84 ± 0.24	17.82 ± 0.18	17.90 ± 0.07
MCHC (g/dL)	34.60 ± 0.94	34.94 ± 0.71	35.14 ± 0.44	35.50 ± 0.58	35.76 ± 0.67	35.52 ± 0.29	35.50 ± 0.19	35.64 ± 0.17
PLT (× 10^3^/µL)	860.20 ± 82.80	852.60 ± 19.71	825.00 ± 28.68	685.40 ± 43.24	674.60 ± 41.33	726.60 ± 64.55	702.40 ± 41.09	618.80 ± 180.06
NEU (%)	26.88 ± 9.72	21.74 ± 6.30	20.04 ± 4.40	22.66 ± 6.45	22.56 ± 3.64	22.94 ± 5.49	22.24 ± 4.25	19.00 ± 3.72
LYM (%)	69.48 ± 9.71	75.06 ± 6.68	76.54 ± 4.80	74.06 ± 6.04	71.52 ± 3.95	72.80 ± 4.81	73.24 ± 4.51	76.32 ± 3.40
MON (%)	1.80 ± 0.46	1.32 ± 0.52	1.60 ± 0.48	1.80 ± 0.44	3.14 ± 0.74	2.20 ± 0.44	1.92 ± 0.62	2.20 ± 0.49
EOS (%)	0.90 ± 0.37	1.18 ± 0.28	1.16 ± 0.23	0.86 ± 0.27	1.56 ± 0.30	1.38 ± 0.41	1.80 ± 0.40	1.56 ± 0.42
BAS (%)	0.28 ± 0.16	0.16 ± 0.09	0.14 ± 0.05	0.18 ± 0.13	0.16 ± 0.05	0.16 ± 0.05	0.32 ± 0.13	0.18 ± 0.08
NEU (× 10^3^/µL)	1.14 ± 0.34	0.76 ± 0.36	0.66 ± 0.18	0.68 ± 0.13	0.68 ± 0.19	0.58 ± 0.08	0.60 ± 0.12	0.44 ± 0.05
LYM (× 10^3^/µL)	3.03 ± 0.69	2.62 ± 0.57	2.47 ± 0.56	2.30 ± 0.58	2.14 ± 0.62	1.94 ± 0.37	1.91 ± 0.37	1.75 ± 0.36
MON (× 10^3^/µL)	0.08 ± 0.02	0.05 ± 0.03	0.05 ± 0.02	0.06 ± 0.02	0.10 ± 0.05	0.06 ± 0.02	0.05 ± 0.02	0.05 ± 0.02
EOS (× 10^3^/µL)	0.04 ± 0.01	0.04 ± 0.01	0.04 ± 0.00	0.02 ± 0.01	0.04 ± 0.01	0.04 ± 0.01	0.05 ± 0.01	0.03 ± 0.01
BAS (× 10^3^/µL)	0.01 ± 0.01	0.00 ± 0.01	0.00 ± 0.00	0.01 ± 0.01	0.00 ± 0.01	0.01 ± 0.01	0.01 ± 0.00	0.00 ± 0.00
RET (%)	3.57 ± 0.35	3.37 ± 0.07	3.35 ± 0.21	3.01 ± 0.29**	2.09 ± 0.35	2.13 ± 0.22	2.27 ± 0.28	2.08 ± 0.10
RETA (× 10^9^/L)	292.82 ± 31.58	276.74 ± 7.63	274.94 ± 13.17	241.06 ± 31.98**	180.18 ± 24.81	184.04 ± 17.98	193.78 ± 22.01	174.62 ± 8.33
APTT (sec)	16.98 ± 0.62	17.04 ± 0.55	17.44 ± 0.57	17.80 ± 0.79	16.43 ± 0.90	16.50 ± 0.69	16.38 ± 0.87	16.72 ± 1.75
PT (sec)	10.20 ± 0.26	10.34 ± 0.09	10.50 ± 0.44	11.46 ± 0.38**	9.95 ± 0.31	10.30 ± 0.19	10.38 ± 0.41	11.20 ± 0.47**

The values are expressed as mean ± SD (n = 5 males and 5 females per group), **Dunnett LSD Test Significant at the 0.01 level.

A significant increase was observed in TCHO of the males in all the exposure groups compared to the control group, whereas no significant changes were observed in the females of the exposure groups and the recovery groups (Table 2).

**Table 2:** Blood chemical parameters of main group male rats exposed to NaDCC.

Sex	Male				Female			
Concentration (mg/m^3^)	0	0.8	4	20	0	0.8	4	20
Na (mmol/L)	142.53 ± 1.32	141.38 ± 1.05	141.78 ± 1.08	140.96 ± 1.08	142.05 ± 0.98	142.58 ± 0.80	142.42 ± 1.60	142.40 ± 0.92
K (mmol/L)	4.60 ± 0.12	4.44 ± 0.17	4.52 ± 0.31	4.28 ± 0.32	4.23 ± 0.25	4.06 ± 0.17	3.96 ± 0.26	4.10 ± 0.25
Cl (mmol/L)	99.78 ± 0.33	100.12 ± 1.01	99.80 ± 0.62	99.44 ± 0.64	102.40 ± 0.93	102.62 ± 0.94	102.84 ± 0.91	103.22 ± 0.84
TP (g/dL)	5.83 ± 0.05	5.74 ± 0.15	5.70 ± 0.12	5.68 ± 0.16	5.85 ± 0.10	5.84 ± 0.05	5.76 ± 0.18	5.76 ± 0.13
ALB (g/dL)	3.98 ± 0.05	3.92 ± 0.04	3.94 ± 0.09	3.90 ± 0.07	3.90 ± 0.00	3.94 ± 0.05	3.88 ± 0.13	3.86 ± 0.09
CREA (mg/dl)	0.41 ± 0.02	0.41 ± 0.03	0.42 ± 0.04	0.41 ± 0.02	0.41 ± 0.02	0.43 ± 0.02	0.42 ± 0.02	0.42 ± 0.02
BUN (mg/dl)	19.83 ± 3.79	20.32 ± 1.82	19.40 ± 2.73	19.62 ± 2.93	19.48 ± 1.20	19.30 ± 2.02	17.72 ± 0.88	18.88 ± 1.80
GLU (mg/dL)	151.13 ± 8.22	156.10 ± 19.44	157.60 ± 13.64	156.72 ± 16.27	143.20 ± 19.74	135.68 ± 19.50	129.22 ± 15.87	123.04 ± 10.64
Ca (mg/dl)	10.15 ± 0.13	10.08 ± 0.15	10.08 ± 0.29	10.12 ± 0.22	9.90 ± 0.16	10.10 ± 0.14	10.04 ± 0.11	9.98 ± 0.13
IP (mg/dl)	8.43 ± 0.43	8.08 ± 0.34	8.18 ± 0.33	8.18 ± 0.60	6.88 ± 0.62	6.74 ± 0.18	6.64 ± 0.35	6.80 ± 0.22
TBIL (mg/dl)	0.18 ± 0.02	0.19 ± 0.01	0.18 ± 0.02	0.19 ± 0.03	0.20 ± 0.02	0.19 ± 0.01	0.19 ± 0.01	0.18 ± 0.01
TCHO (mg/dl)	56.10 ± 3.97	66.68 ± 3.00*	67.72 ± 2.71**	65.64 ± 7.79**	95.80 ± 4.62	92.04 ± 5.22	92.86 ± 4.98	91.18 ± 4.99
TG (mg/dl)	53.18 ± 20.87	50.76 ± 7.21	56.96 ± 14.94	35.72 ± 14.48	77.38 ± 35.26	56.56 ± 18.35	70.24 ± 33.07	41.48 ± 22.01
AST (IU/L)	79.90 ± 5.95	81.88 ± 11.80	84.36 ± 8.80	86.44 ± 7.39	75.50 ± 11.26	74.36 ± 6.58	76.36 ± 4.05	82.04 ± 6.39
ALT (IU/L)	60.45 ± 2.74	54.94 ± 7.25	68.96 ± 9.74	55.74 ± 6.00	58.15 ± 4.18	58.00 ± 5.82	57.90 ± 3.63	57.06 ± 7.43
ALP (IU/L)	1022.95 ± 111.05	985.30 ± 36.53	949.58 ± 39.28	949.00 ± 48.75	575.15 ± 52.79	564.84 ± 35.50	574.76 ± 63.59	622.76 ± 22.23
LDH (IU/L)	902.55 ± 289.03	1067.26 ± 591.82	996.78 ± 500.82	856.36 ± 420.07	570.73 ± 364.81	448.36 ± 249.98	439.80 ± 184.22	444.62 ± 250.48
CPK (U/L)	315.53 ± 77.43	367.76 ± 152.87	351.18 ± 133.88	294.46 ± 95.01	207.53 ± 83.46	177.58 ± 66.55	174.92 ± 43.49	179.40 ± 58.30
A/G ratio	2.15 ± 0.06	2.18 ± 0.11	2.24 ± 0.09	2.20 ± 0.10	1.98 ± 0.15	2.10 ± 0.00	2.08 ± 0.08	2.04 ± 0.09

The values are expressed as mean ± SD (n = 5 males per group), *Dunnett Test Significant at the 0.05 level; **Dunnett Test Significant at the 0.01 level.

**Figure 4:**
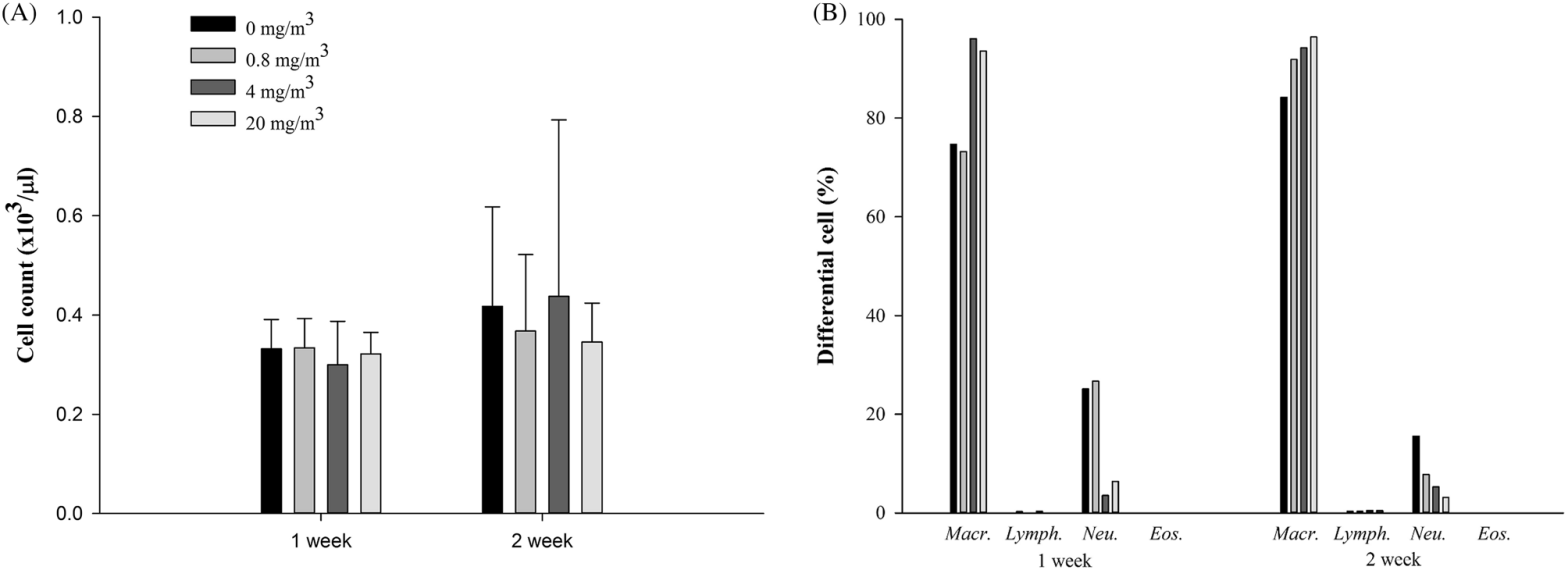
Total cell counts (**A**) and differential cell percentage (**B**) of total cells from bronchoalveolar lavage fluid (BALF) after NaDCC exposure. The values are expressed as mean ± SD (n = 5 males per group).

**Figure 5:**
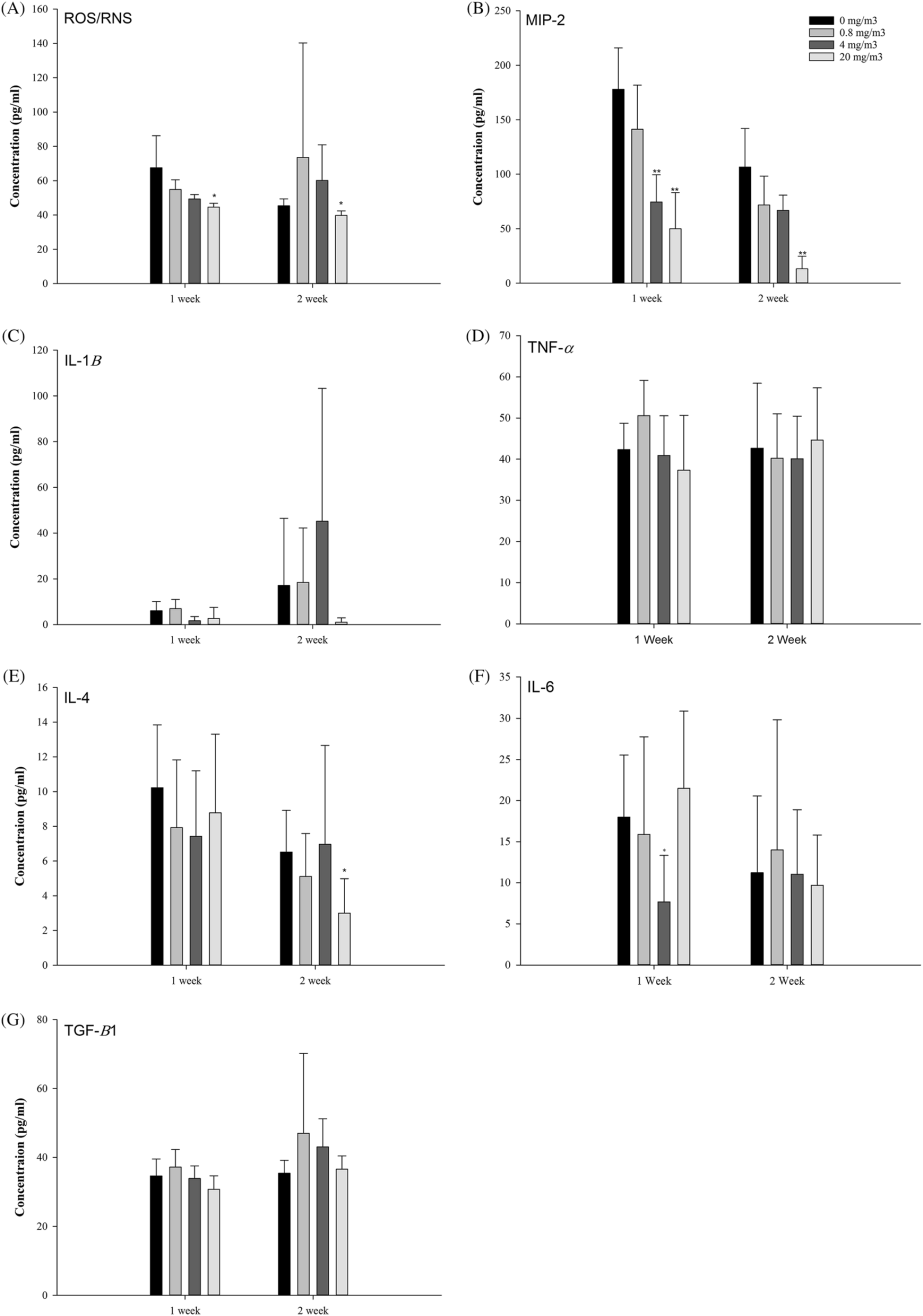
Concentrations of cytokines in bronchoalveolar lavage fluid (**A**–**G**). The values are expressed as mean ± SD (n = 5 males per group). Significantly different from control by Dunn Rank Sum test: *p < 0.05; **p < 0.001.

Examination of the BALF at 1 and 2 weeks after exposure to the test substance did not reveal any significant change in any of the exposure groups, although there was an increase in the number of macrophages and a decrease in the number of neutrophils (Fig. 4). The concentrations of ROS/RNS and MIP-2 showed a concentration-dependent decrease in the test-substance-exposure groups. Although IL-1β tended to decrease in the test-substance-exposure groups, no changes were observed in TNF-α, IL-4, IL-6, and TGF-β levels (Fig. 5).

Significant changes were found in the absolute and relative weight of the heart and the absolute weight of the spleen in the males exposed to 20 mg/m^3^ compared to those in the control group. In the females exposed to 20 mg/m^3^, a significant change was observed in the relative weight of the brain compared to that in the control group. There were no significant changes in the organs of the animals in the recovery groups (Table 3).

**Table 3:** Absolute and relative organ weights of main group rats exposed to NaDCC.

Sex	Male				Female			
Concentration (mg/m^3^)	0	0.8	4	20	0	0.8	4	20
**Absolute organ weight (g)**								
Brain	1.77 ± 0.05	1.79 ± 0.01	1.76 ± 0.03	1.71 ± 0.02	1.67 ± 0.07	1.72 ± 0.04	1.69 ± 0.05	1.70 ± 0.03
Heart	0.61 ± 0.02	0.60 ± 0.01	0.58 ± 0.03	0.57 ± 0.03*	0.50 ± 0.03	0.49 ± 0.03	0.48 ± 0.03	0.48 ± 0.02
Lung	0.38 ± 0.05	0.37 ± 0.01	0.36 ± 0.02	0.37 ± 0.04	0.30 ± 0.02	0.31 ± 0.01	0.30 ± 0.02	0.30 ± 0.02
Liver	5.84 ± 0.43	5.42 ± 0.20	5.46 ± 0.42	4.85 ± 0.52	4.02 ± 0.11	3.98 ± 0.17	3.85 ± 0.24	3.74 ± 0.12
Spleen	0.47 ± 0.02	0.44 ± 0.03	0.43 ± 0.04	0.39 ± 0.03**	0.36 ± 0.03	0.34 ± 0.01	0.34 ± 0.02	0.33 ± 0.01
Kidney	1.34 ± 0.08	1.32 ± 0.05	1.29 ± 0.04	1.20 ± 0.08	1.04 ± 0.03	1.04 ± 0.03	1.01 ± 0.06	1.02 ± 0.03
**Relative organ weight (%)**								
Brain	0.94 ± 0.04	1.00 ± 0.05	1.00 ± 0.06	1.05 ± 0.08	1.12 ± 0.04	1.15 ± 0.02	1.16 ± 0.05	1.21 ± 0.05^#^
Heart	0.32 ± 0.01	0.34 ± 0.01	0.33 ± 0.01	0.35 ± 0.01*	0.33 ± 0.02	0.33 ± 0.02	0.33 ± 0.02	0.34 ± 0.02
Lung	0.20 ± 0.03	0.21 ± 0.01	0.20 ± 0.01	0.22 ± 0.01	0.20 ± 0.01	0.21 ± 0.01	0.21 ± 0.01	0.21 ± 0.02
Liver	3.09 ± 0.10	3.02 ± 0.04	3.07 ± 0.13	2.97 ± 0.16	2.69 ± 0.08	2.68 ± 0.14	2.64 ± 0.11	2.66 ± 0.11
Spleen	0.25 ± 0.01	0.24 ± 0.01	0.24 ± 0.02	0.24 ± 0.01	0.24 ± 0.02	0.23 ± 0.02	0.23 ± 0.01	0.24 ± 0.01
Kidney	0.71 ± 0.02	0.74 ± 0.03	0.73 ± 0.03	0.74 ± 0.05	0.70 ± 0.02	0.70 ± 0.02	0.69 ± 0.02	0.73 ± 0.02

The values are expressed as mean ± SD (n = 5 males and 5 females per group).

*Dunnett LSD Test Significant at the 0.05 level; **Dunnett LSD Test Significant at the 0.01 level.

^#^ = Dunn Rank Sum Test Significant at the 0.05 level.

**Figure 6:**
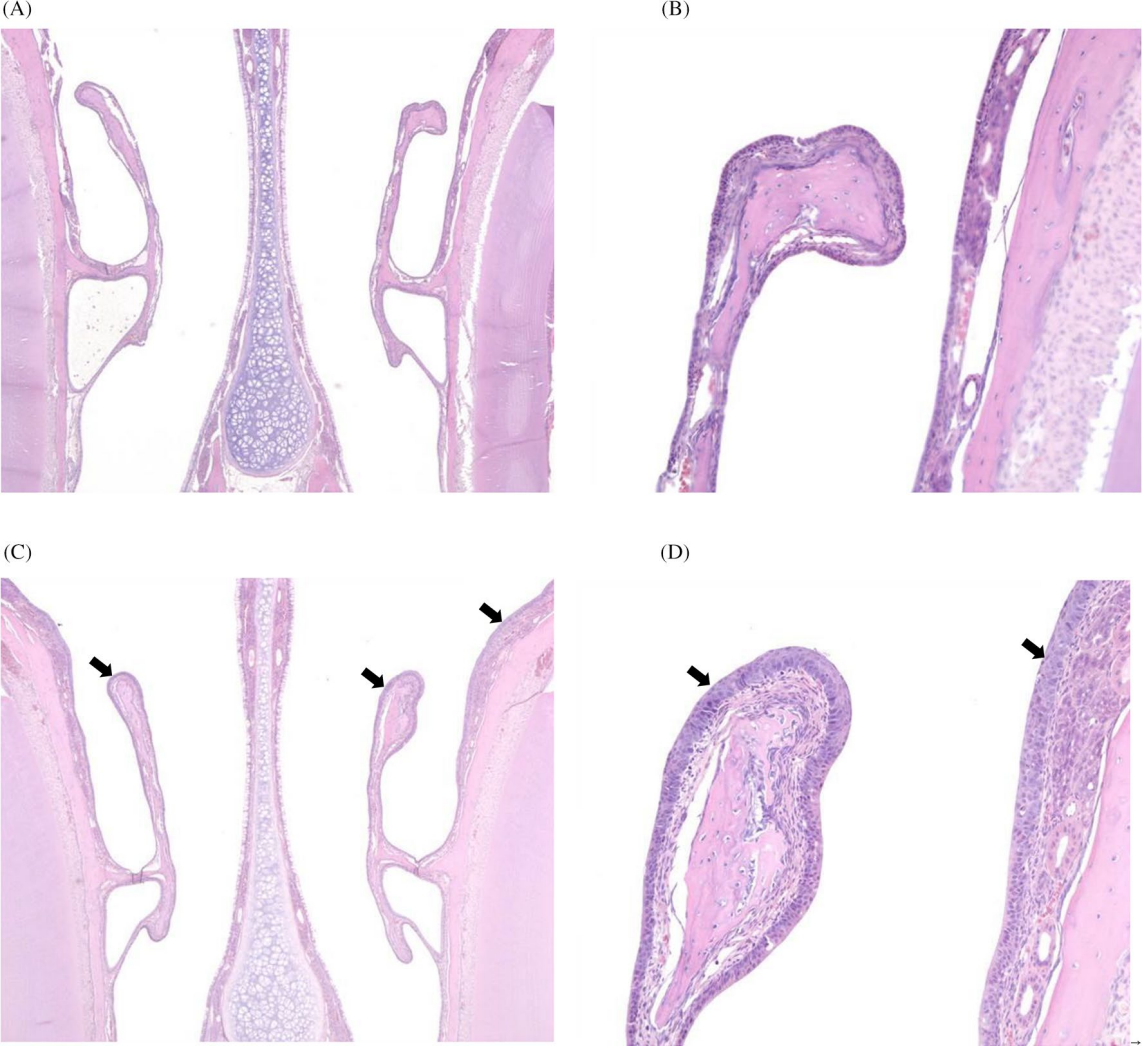
Histopathology of the nasal cavity of male rats exposed to NaDCC, (**A**, **B**) No abnormal lesion was observed In the control group, (**C**, **D**) Degeneration (arrow) of transitional epithelium was observed in the 20 mg/m^3^ exposed group. (**A**, **C**) × 50, (**B**, **D**) × 400, respectively, HE.

**Figure 7:**
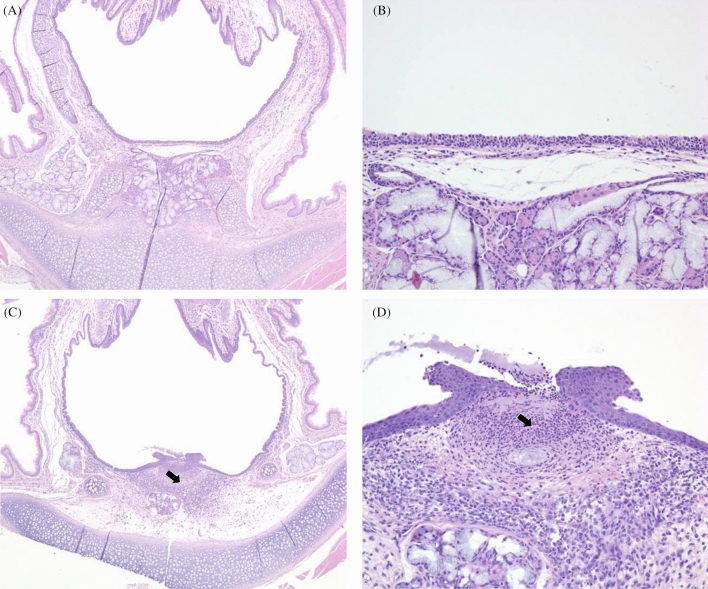
Histopathology of the larynx of male rats exposed to NaDCC, (**A**, **B**) No abnormal lesion was observed in the control group, (**C**, **D**) Inflammation (arrow) of epithelium was observed in the 20 mg/m^3^ exposed group. (**A**, **C**) × 50, (**B**, **D**) × 400, respectively, HE.

The autopsy did not reveal any findings related to the exposure to the test substance in any animal. Histopathological examination was performed to examine the effects of test substance exposure in the nasal cavity and larynx in both males and females of the test substance exposure groups (Table 4, Figs. 6, 7). In the males of the 20-mg/m^3^-exposure group, we observed a reduction in respiratory epithelium goblet cells and hypertrophy of other cells in the respiratory epithelium, degeneration and/or hyperplasia of the transitional epithelium in the nasal cavity, inflammation and ulceration of the epithelial cells, and squamous metaplasia in the larynx. Epithelial cell inflammation was observed in the larynx of the males in the 4-mg/m^3^-exposure group. In addition, mineralization was observed in the corticomedullary junction of the kidney in the females exposed to 20 mg/m^3^ of the test substance.

**Table 4:** Histopathological assessment of the nasal cavity tissues and larynix in rats.

	Main group								Recovery group
Sex	Male				Female				Male
Concentration (mg/m^3^)	0	0.8	4	20	0	0.8	4	20	20
**Nasal cavity**									
Number of animals	5	5	5	5	5	5	5	5	5
Decreased goblet cells, respiratory epithelium	(0)	(0)	(0)	(2)	(0)	(0)	(0)	(0)	(0)
Minimal	0	0	0	2	0	0	0	0	0
Mean ± SD	0.00 ± 0.00	0.00 ± 0.00	0.00 ± 0.00	0.40 ± 0.55	0.00 ± 0.00	0.00 ± 0.00	0.00 ± 0.00	0.00 ± 0.00	0.00 ± 0.00
Hypertrophy, goblet cells, respiratory epithelium	(0)	(0)	(0)	(1)	(0)	(0)	(0)	(0)	(0)
Minimal	0	0	0	1	0	0	0	0	0
Mean ± SD	0.00 ± 0.00	0.00 ± 0.00	0.00 ± 0.00	0.20 ± 0.45	0.00 ± 0.00	0.00 ± 0.00	0.00 ± 0.00	0.00 ± 0.00	0.00 ± 0.00
Degeneration, transitional epithelium	(0)	(0)	(0)	(4)	(0)	(0)	(0)	(4)	(0)
Moderate	0	0	0	2	0	0	0	2	0
Marked	0	0	0	2	0	0	0	2	0
Mean ± SD	0.00 ± 0.00	0.00 ± 0.00	0.00 ± 0.00	2.80 ± 1.64	0.00 ± 0.00	0.00 ± 0.00	0.00 ± 0.00	2.80 ± 1.64	0.00 ± 0.00
Hyperplasia, transitional epithelium, focal	(0)	(0)	(0)	(1)	(0)	(0)	(0)	(1)	(0)0
Mild	0	0	0	1	0	0	0	1	0
Mean ± SD	0.00 ± 0.00	0.00 ± 0.00	0.00 ± 0.00	0.40 ± 0.89	0.00 ± 0.00	0.00 ± 0.00	0.00 ± 0.00	0.40 ± 0.89	0.00 ± 0.00
**Larynx**									
Number of animals	5	5	5	5	5	5	5	5	5
Inflammation, mixed, epithelium	(0)	(0)	(1)	(1)	(0)	(0)	(0)	(2)	(0)
Minimal	0	0	1	0	0	0	0	1	0
Mild	0	0	0	1	0	0	0	0	0
Moderate	0	0	0	0	0	0	0	1	0
Mean ± SD	0.00 ± 0.00	0.00 ± 0.00	0.20 ± 0.45	0.40 ± 0.89	0.00 ± 0.00	0.00 ± 0.00	0.00 ± 0.00	0.80 ± 1.30	0.00 ± 0.00
Ulceration, epithelium	(0)	(0)	(0)	(1)	(0)	(0)	(0)	(0)	(0)
Minimal	0	0	0	1	0	0	0	0	0
Mean ± SD	0.00 ± 0.00	0.00 ± 0.00	0.00 ± 0.00	0.00 ± 0.45	0.00 ± 0.00	0.00 ± 0.00	0.00 ± 0.00	0.00 ± 0.00	0.00 ± 0.00
Squmous metaplasia, epithelium	(0)	(0)	(0)	(2)	(0)	(0)	(0)	(2)	(0)
Minimal	0	0	0	2	0	0	0	1	0
Mild	0	0	0	0	0	0	0	1	0
Mean ± SD	0.00 ± 0.00	0.00 ± 0.00	0.20 ± 0.45	0.40 ± 0.55	0.00 ± 0.00	0.00 ± 0.00	0.00 ± 0.00	0.60 ± 0.89	0.00 ± 0.00

0: unremarkable = no presence of histopathologic lesion; 1: minimal = lesions involving < 10% of the tissue of each organ; 2: mild = lesions involving < 10–30% of the tissue of each organ; 3: moderate = lesions involving < 30–50% of the tissue of each organ; 4: marked = lesions involving < 50–70% of the tissue of each organ; 5: severe = lesions involving > 70% of the tissue of each organ.

## Discussion

In this study, whole-body inhalation exposure was performed for 6 h a day for 14 days at exposure concentrations of 0.8, 4, and 20 mg/m^3^ in F344 rats to evaluate the response of repeated exposure to NaDCC. In addition, a two-week recovery period was used to evaluate the reversibility of toxicity.

The average concentration in the chamber measured during the exposure period of the test substance was < 20%, which satisfies the conditions for aerosol generation in OECD TG412^17^. This was thought to be due to the increase in compressed air pressure to increase the concentration of the test substance in the chamber when the test substance was generated^18^.

Weight loss and food intake reduction were observed in both males and females in the 20-mg/m^3^-exposure and recovery groups at 2 weeks after exposure. It is judged that the weight was decreased due to the decrease in feed intake due to the irritating effect of the exposure of the test substance.

The decrease in WBC counts in males of 4- and 20-mg/m^3^-exposure groups was attributed to the observed weight loss accompanied by dehydration. Similarly, the decrease in hematopoietic function as evidenced by increase in PT in both males and females of the 20-mg/m^3^-exposure group was also due to the weight loss and decrease in food intake^19,20^. The observed increase in BASO in the females of the 4-mg/m^3^-exposure group had no dose-dependence or statistical significance and was not judged to have any toxicological significance. The increase in TCHO observed in the males of the test substance exposure group had no toxicological significance as the changes were within the normal range.

Although BALF analysis showed statistically significant decreases in neutrophils, ROS/RNS and MIP-2 after 1 week of exposure to the test substance, the concentration-dependent decrease was observed, and the test substance-related effects in the histopathological findings were observed only in the nasal cavity and bronchi, but it was not observed in the lungs. Therefore, it is not judged as an effect related to exposure to the test substance, but it is considered that additional investigation is necessary for the observation that neutrophils were higher than our background value (< 1%) in all test groups.

In the 20-mg/m^3^-exposed group, we observed a decrease in heart and spleen weights in males and an increase in brain weights in females. However, as these changes could be attributed to the observed weight loss in the animals, and there were no specific findings in the histopathological examination, the changes were not considered to be toxicologically significant.

In histopathological examination, test substance-related findings were observed in the nasal cavity and/or larynx of the 4- and 20-mg/m^3^-exposed groups. NaDCC, a test substance, is known to cause irritation to the skin and eyes (https://echa.europa.eu/registration-dossier/-/registered-dossier/14822/7/4/1). The NIOSH reported that TCCA and NaDCC are extremely irritating at relatively low concentrations and that their potential for causing serious injury to the respiratory system should not be underestimated^11^. Therefore, these findings induced in the nasal cavity and larynx were considered to be caused by irritation caused by exposure to the test substance.

From the above results, the NOAEC was evaluated as 0.8 mg/m^3^ under the conditions of this test, and based on these results, DNEL was evaluated as 0.016 mg/m^3^ by applying an interspecies factor of 2.5, intraspecies factor of 5, and exposure duration factor of 4 as default assessment factors^21^.

In conclusion, the findings of this study confirmed that exposure to NaDCC by repeated inhalation for 2 weeks mainly affected the upper respiratory tract: nasal cavity and larynx. Exposure-related effects of the test substance were observed even at the exposure concentration of 4 mg/m^3^, and the No Observed Adverse Effects Concentration (NOAEC) was considered to be 0.8-mg/m^3^. Moreover, since these test substance-related effects were not observed in the recovery group, they were evaluated as reversible responses. From these results, DNEL was evaluated as 0.016 mg/m^3^. These results can be used as reference data for long-term exposure toxicity studies and as basic data for protecting workers' health at NaDCC-treated workplaces and identifying causes of accidents with humidifier disinfectants.

## Materials and methods

### Test chemical

NaDCC (both the dihydrate and anhydrous material), as well as cyanuric acid, are well-characterized substances. Physical and chemical properties are described in the Kirk–Othmer Encyclopedia of Chemical Technology^22^, in a web-based document on chloroisocyanurates by Occidental Chemical Corporation^23,24^, in a monograph developed by OxyChem on the chemistry of the chloroisocyanurates^25^, and in a Food Additive Petition (FAP) submitted by Occidental to the U.S. Food and Drug Administration^17^. NaDCC was purchased from Sigma-Aldrich (Saint Louis, USA).

### Experimental design

NaDCC used in humidifier disinfectants in the living environment has an exposure concentration of 0.1–0.2 mg/m^3^ (1–2 tablets a day, 4.53% per tablet, 320 mg per tablet, 24 h of use, average volume of use of 30.3 m^3^, and winter average ventilation of 0.2 times/h). According to the "Humidifier Disinfectant-Containing Substance (NaDCC) Inhalation Toxicity Study (Publication No. 11-1480523-003846-01)" of the National Institute of Environmental Research in Korea, five (5/5) males and two (2/5) females died at the exposure concentration of 250-mg/m^3^ of NaDCC in an acute inhalation toxicity test using 344 rats. At the exposure concentration of 40-mg/m^3^, five (5/5) males and one (1/5) females died. Therefore, it can be inferred that NaDCC contains 4–8 times the concentration ranging from 0.1 to 0.2-mg/m^3^. In order to confirm certain toxicity, we set 20-mg/m^3^ as the high concentration of exposure and applied a common ratio 5 to set 4- and 0.8-mg/m^3^ as the medium and low concentrations, respectively. In addition, since it was judged that the toxicity would be more strongly induced in male animals, an interim test group for bronchoalveolar fluid (BALF) examination 7 days after exposure and a recovery test group for the evaluation of the presence or absence of reversibility of toxicity were also assigned for males. The exposure period of the test substance was set at 6 h a day, 5 days a week for 14 days, and the recovery period was for 14 days after the end of exposure.

### Exposure and analysis

For exposure to the test substance, NaDCC was dispersed in water (however, the test substance was not observed with the naked eye), and an aerosol was generated using an atomizer-type mist generator (NB-2N, Sibata Co. Ltd., Japan). The aerosol generated to maintain the target concentration in the chamber was diluted with air from the Aerosol Dilution System and supplied to the whole body chamber (1.4 m^3^). The control group was supplied only clean air without test substances, but other environmental conditions in the chamber were the same for both control and test exposure groups.

Samples of the test substance in the chamber were collected three times using a 25-mm micro-glass filter and a personal sample collector (Model No. Airchek XR 5000, SKC Inc., USA) from the breathing area of the test animals during exposure to the test substance. The weight of the filter with the test substance collected was measured using an electronic balance (Model No. 770-60, KERN & SOHN GmbH Co. Ltd., German). The concentration of the test substance in the chamber was calculated by measuring the filter weight before and after collection. When measuring the weight of the filter, it was measured by excluding the influence of moisture. In addition, while the test substance aerosol was being generated, the number of aerosol particles was checked in real time using a Portable Aerosol Spectrometer (Model 1.109, GRIMM Aerosol Technik GmbH & Co.KG, Germany). The mass median aerodynamic diameter (MMAD) and geometric standard deviation (GSD) were determined for each exposure concentration using a Cascade impactor (Model 135, MiniMOUDI Impactor, MSP Co. LTD., USA) during exposure to the test substance to confirm the particle size distribution of the aerosols.

### Test system

In this study, the F344 rat was selected as the test system. This strain was selected because of the abundance of basic data comparable to toxicity through inhalation exposure. F344 rats (6 weeks of age, Specific Pathogen Free animal) were purchased from Japan SLC, Inc. (Shizuoka, Japan); on the day of obtaining the animals, all animals were weighed using an electronic balance (QUINTIX3102-1SKR, Sartorius, Germany). The males weighed 83.94–118.22 g, and the females weighed 102.35–124.41 g. Clinical signs were recorded on the day the animals were obtained. The rats were allowed to acclimate to their housing environment and quarantined for 7 days; no abnormality was observed in any animal.

Based on the weight of the animals, the test animals were allocated to 4 test groups-5 animals per group-such that the average weight of all the groups was the same. In addition, the interim and recovery groups were also formed in the same way as the test groups. During the study period, ≤ 3 rats were housed in a polysulfone cage (W 310 × L 500 × H 200 mm), but the rats were housed individually in a 6-wire mesh cage (W 240 × L 1200 × H 200 mm) during the period of exposure. During the exposure time of the test substance, feed was not supplied, but water was supplied. The animal room conditions were as follows: temperature of 19.0–25 °C, humidity of 30–70%, light/dark cycle of 12 h/day, illuminance of 150–300 Lux, and ventilation frequency of 10–20 times/h. This study was carried out in compliance with the Arrive guidelines (https://arriveguidelines.org). The protocol of this study was approved by the Institutional Animal Care and Use Committee (IACUC) of Occupational Safety and Health Research Institute in March 2019 (Approval Number IACUC-1922). This study conducted general welfare for animals according to the Standard Operating Procedure (SOP) of the Inhalation Toxicity Study Center, Occupational Safety and Health Research Institute, and was conducted in accordance with the Guide for the Care and Use of Laboratory Animals (by ILAR publication).

### Observations, analysis, and pathological examination

On the day of necropsy, all surviving animals were sampled under inhalation anesthesia with isoflurane to reduce animal suffering. Isoflurane used in this study is the recommended anesthetic for laboratory mice as it provides a high safety margin with acceptable side effects^26^. After blood collection, the abdominal arteries and veins were cut and exsanguinated, and the gross examination was performed. The sampled blood was subjected to hematological and blood biochemical analyses. The following hematological parameters were evaluated with an automatic blood cell automatic analyzer (ADVIA 2120i, SIEMENS, Germany) and an automatic coagulation time meter (Coapresta 2000, SEKISUI, Japan).

**Table:** 

Leucocyte (WBC)^a^	WBC differential count^a,d^
Erythrocyte count (RBC)^a^	Neutrophils (NEU)
Hemoglobin (Hb)^a^	Lymphocytes (LYM)
Hematocrit (HCT)^a^	Monocytes (MON)
Mean corpuscular volume (MCV)^a^	Eosinophils (EOS)
Mean corpuscular hemoglobin (MCH)^a^	Basophils (BAS)
Mean corpuscular hemoglobin concentration (MCHC)^a^	Activated partial thromboplastin time (APTT)^b^
Platelet count (PLT)^a^	Prothrombin time (PT)^b^
Reticulocyte count (RET)^a,c^	

^a^Measured by using an ADVIA2120i hematology analyzer (Siemens, Germany).

^b^Measured by using an ELITE coagulation analyzer (Instrumentation Laboratory, USA).

^c^Absolute (A) and relative (%) counts.

^d^Absolute (A) and relative (%) differential counts.

The following blood biochemical parameters were measured with an automatic analyzer (TBA-120FR NEO, Toshiba Co., Japan).

**Table:** 

Sodium (Na)	Total bilirubin (TBIL)
Potassium (K)	Total cholesterol (TCHO)
Chloride (Cl)	Triglyceride (TG)
Total protein (TP)	Aspartate aminotransferase (AST)
Albumin (ALB)	Alanine aminotransferase (ALT)
Creatinine (CREA)	Alkaline phosphatase (ALP)
Blood urea nitrogen (BUN)	γ-glutamyl transpeptidase (γ-GTP)
Glucose (GLU)	lactate dehydrogenase (LDH)
Calcium (Ca)	creatinine phosphokinase (CPK)
Inorganic phosphorus (IP)	Albumin/globulin (A/G ratio)

BALF of only male rats was analyzed 1 and 2 weeks after exposure. To obtain BALF, the upper end of the trachea was cut, and a polypropylene tube attached to a syringe was inserted; the trachea was then washed three times with 4 mL of phosphate-buffered saline (PBS). The collected BALF was centrifuged at 450*g* for 10 min, and the supernatant was stored at − 80 °C. The cell pellet was re-suspended in fresh PBS, and the total immune cell count was determined by using a Hematology Analyzer (ADVIA 2120i). The re-suspended cell pellet was centrifuged at 270*g* for 10 min using a Cytospin centrifuge (Cellspin; Hanil, Gimpo, Korea) and stained using Diff-Quick staining solution. Differential cell counts were determined using a light microscope at 100 × magnification.

The supernatant separated from the BALF was thawed at ~ 20 °C just before cytokine analysis. A commercially available cytokine multi-magnetic bead array kit (R&D Systems, Minneapolis, MN 55413) was used to determine the concentrations of interleukin (IL)-1β, IL-6, IL-4, tumor necrosis factor alpha (TNF-α), and macrophage inflammatory protein 2-alpha (MIP-2) in the BALF. The Magnetic Bead Single Plex Kit (MILLIPLEX MAP; Merck Millipore, Darmstadt, Germany) was used to measure the concentration of transforming growth factor β (TGF-β). Reactive oxygen species (ROS)/reactive nitrogen species (RNS) was analyzed using an OxiSelect™ In Vitro ROS/RNS Assay Kit (Catalog No. STA-347; Cell Biolab, Inc., USA) and Varioskan Flash Reader (Thermo Fisher Scientific, Finland). The assays were performed per the manufacturers' instructions. The median fluorescence intensity of the samples was measured using a Luminex 100 instrument (Luminex, Austin, TX, USA), and standard curves were obtained using MasterPlex software (MasterPlex QT 2010; Miraibio, Hitachi, CA, USA). Cytokine concentrations were calculated using the standard curves.

The following organs of all animals were harvested and the absolute and relative (organ-to-body weight ratios) weights were measured: brain, liver, heart, spleen, lung, and kidneys. Bilateral organs were weighed together. Histopathological examination was performed by making tissue samples for the organs of the control- and the high-concentration groups and the organs of the low- and medium- concentration groups, which are predicted to change due to the test substance, as follows.

**Table:** 

Control- and high-concentration groups		Low- and medium-concentration groups	
Brain	Lung	Nasal cavity	Testis
Heart	Nasal cavity	Larynx	Epididymis
Liver	Larynx	Trachea	Prostate
Spleen	Trachea	Liver	Seminal vesicles
Kidney		Spleen	Uterus
		Kidney	Vaginal organs
		Thymus	

### Statistical analysis

The data obtained during the study period are presented as means and standard deviation values. The data were statistically analyzed using PRISTIMA version 7.1.0 (Xybion Medical Systems Corporation, Morris Plains, NJ, USA). Levene’s test was performed to determine the homogeneity of the variances. When variances were homogeneous, one-way analysis of variance (ANOVA) was performed, and statistical differences between the control and exposed groups were analyzed by Dunnett’s test. When variances were not homogeneous, Kruskal–Wallis test was performed, and statistical differences between the control and exposure groups were analyzed by Dunn’s rank sum test.

### Ethics approval and consent to participate

The Institute for Occupational Safety and Health was certified by AAALAC International (Association for Assessment and Accreditation of Laboratory Animal Care International) in 2018. This study plan has been reviewed by the Institutional Animal Care and Use Committee (IACUC). This study was carried out in accordance with the standard operating procedure of the Institute of Occupational Safety and Health and the study plan. All attempts to ensure the general welfare of animals were carried out. The animal study was carried out according to the Animal Protection Law and the Guide for the Care and Use of Laboratory Animal.

### Consent for publication

Consent for publication was obtained.

## Data Availability

All data will be made available to those who make a justified request.
