# Author Correction: Humidifier disinfectant, sodium dichloroisocyanurate (NaDCC): assessment of respiratory effects to protect workers’ health

**DOI:** 10.1038/s41598-024-59792-z

**Published:** 2024-04-19

**Authors:** DongSeok Seo, Yong-Hoon Lee, JiMin Jo

**Affiliations:** Inhalation Toxicity Study Center, Occupational Safety and Health Research Institute, Korea Occupational Safety and Health Agency, 30, Expro-ro 339beon-gil, Yuseong-gu, Daejeon, Republic of Korea

Correction to: *Scientific Reports* 10.1038/s41598-021-95148-7, published online 03 August 2021

Yong-Hoon Lee was omitted from the author list in the original version of this Article.

The Author Contributions section now reads:

“S.D.S. designed this study and collected and analysed data on disinfectants. Y.H.L conducted the pathological analyses of animals. J.M.J. established exposure condition and performed particle exposure and characterization. The authors read and approved the final manuscript.”

The original Article has been corrected.

